# A feasibility (pilot) mixed methods study of an innovative non-pharmacological breath-based yoga and social-emotional intervention program in an at-risk youth sample in London, Canada

**DOI:** 10.1186/s40814-024-01452-0

**Published:** 2024-02-07

**Authors:** Akshya Vasudev, Emily Ionson, Janani Sathiaselan, Anurag Thatipalli, Aman Chauhan, Christine Hassan, Javeed Sukhera, Mark Speechley, Cheryl Forchuk

**Affiliations:** 1 Present Address: Integrative Psychiatry Lab, Lawson Health Research Institute and Parkwood Institute of Research, Parkwood Mental Health Building, Parkwood Hospital, London, ON Canada; 2 Department of Psychiatry, Schulich School of Medicine and Dentistry, Western University, and London Health Sciences Centre, London, ON Canada; 3 Department of Epidemiology and Biostatistics, Western University, London, ON Canada

**Keywords:** Youth at-risk, Homeless, Mental health, Depression, Yoga

## Abstract

**Background:**

Various service provision models for youth at risk of homelessness have been researched and implemented, including access to housing and physical and mental health resources. However, even with these interventions, we remain unaware of how best to manage symptoms of depression and anxiety and the rate of drug use in these populations primarily because of a lack of feasibility data.

**Methods:**

This paper presents the results of a mixed-methods study in London, Canada, that examined the feasibility of implementing a biopsychosocial intervention, SKY Schools, in at-risk youth aged between 16 and 25 (*n* = 49). The study also recorded qualitative responses about the program’s usefulness from the perspective of the service users. The SKY Schools intervention consisted of social-emotional learning combined with Sudarshan Kriya Yoga, a standardized yoga-based breathing exercise routine. The intervention program was divided into two phases: an active learning phase and a reinforcement phase. The following feasibility outcome measures were collected: (1) the number of potential participants approached per month, (2) number (proportion) who were successfully screened, (3) the proportion of screened participants who enrolled, (4) the rate of retention in the study, (5) rate of adherence to study protocol, (6) proportion of planned ratings that were completed, (7) intervention cost per case, (8) completeness of final data for analysis, (9) length of time to collect all data, (10) quality of all collected data, (11) determining if partnering community organizations were willing to conduct the study as per study protocol, (12) determining if there were any capacity issues with partners providing intervention and investigators being able to perform the tasks that they were committed to doing, (13) determining if there were any problems of entering the data into a computer, (14) preliminary data about the safety of the intervention, and (15) preliminary estimate of treatment effects.

**Results:**

All feasibility outcome measures were collectible. In the city of London, Canada it was feasible to conduct a pilot study in this population of youth at risk of homelessness. Foremost among the findings was a high retention rate (61.2%) and overall positive qualitative feedback with a number of potential suggestions to improve the delivery and quality of the intervention. However, we had a significantly low recruitment rate (0.27 participants per week) suggesting that multiple sites will be needed to achieve an adequate sample size for a subsequent definitive trial.

**Conclusions:**

Future researchers may consider the findings of this feasibility study when designing a randomized control trial to further assess the efficacy and tolerability of SKY Schools.

**Trial registration:**

Trial registration: Clinicaltrials.gov, identifier NCT02749240. Registered April 22, 2016, https://clinicaltrials.gov/ct2/show/NCT02749240.

**Supplementary Information:**

The online version contains supplementary material available at 10.1186/s40814-024-01452-0.

## Key messages regarding feasibility

1) What uncertainties existed regarding the feasibility?

It is extremely difficult to conduct clinical intervention trials in youth at risk of homelessness, although they are at the highest risk of depression, anxiety, and substance use. Hence, collecting quantitative and qualitative data from this sample is important to inform future studies.

2) What are the key feasibility findings?

Using a biopsychosocial intervention designed specifically for youth at risk of homelessness, we were able to retain 61% of the study sample. Mixed messaging was obtained regarding the intervention’s benefit and the trial delivery logistics.

3) What are the implications of the feasibility findings for the design of the main study?

Our findings confirm that youth at risk of homelessness are a very difficult population to recruit and retain for the purpose of running a clinical trial. Our findings also suggest that there is a need for improved content as well as delivery of the intervention; specifically, excluding those who are already homeless as they are unable to receive the intervention consistently and providing better environmental and child care facilities.

## Background

Youth at risk of homelessness are best defined as those who are highly susceptible to becoming homeless due to economic, personal, or familial situations [1]. This group is likely one of the most vulnerable members of society. In a sample of such youth from downtown Toronto, Canada, it was found that the majority had left home and school prematurely, had been arrested in their lifetime, and used at least one illicit drug in the past 12 months. A substantial number had been imprisoned, experienced physical abuse, and exhibited depressive symptomatology and suicidal ideation [2]. Similar results were found in another metropolitan city, Vancouver, Canada [3] with high rates of incarceration as well as stimulant and opioid use.

Various service provision models have been considered for this population, yet there is a lack of a gold-standard model, likely due to difficulty in conducting clinical trials in this population. Expert opinion based on small trial data suggests that supported housing, transitional case management, and collaborative care models are useful [4]. In this study, a Delphi survey was conducted on experts in the field of at-risk populations. They suggested the need for prioritization of the provision of mental health and addiction care, facilitating access to permanent housing and income support, and case management/care coordination. The survey participants also ranked specific homeless subpopulations in need of additional research including Indigenous Peoples (First Nations, Métis, and Inuit),youth; women and families; people with acquired brain injury, intellectual or physical disabilities; and refugees and other migrants [4].

Within the field of mental health care delivery, recent trend data about the services for youth in the Canadian population suggest a strong increase in past-year mental health consultations from 2011 to 2018, a rise in the prevalence of diagnosed mood and anxiety disorders, and a slight increase in illicit drug use [5]. In the Canadian province of Ontario, the rates of mental health emergency department visits among youth increased from 11.7 per 1000 in 2003 to 24.1 per 1000 in 2017 [6]. Finally, a recent scorecard system to assess issues related to access and quality of mental health and addiction systems in Ontario showed that the highest rates of deliberate self-harm and the greatest rise over time in overall mental health and addiction-related outpatient visits, emergency department visits, and hospitalizations were experienced by individuals aged 14–24 years [7]. Taking the above data together, it could be that traditional hospital-based youth mental health services are currently unable to adequately manage youth mental health services in Ontario. While data are not available, it could be that further marginalized youth such as those at risk of homelessness, who are the most in need of services, are unable to access traditional psychiatric services for various reasons including stigma and system pressures.

Being aware of these pressures, our team has previously attempted to design an integrative and collaborative youth mental health service model in a medium-sized Canadian city (London, Ontario). We have previously partnered with Youth Opportunities Unlimited (YOU), which started as a downtown transition home established in 1982 with dedicated funding from various federal and provincial support organizations. Approximately 3600 youth between the ages of 16–30 access the system of support available at YOU each year. Services have expanded over the years and include the provision of basic needs and housing; access to physical, mental, and dental health care; education and employment support; and social learning opportunities designed to help youth lead positive lives [8]. The agency has numerous service programs including a drop-in center with meals, counseling, social enterprises for employment, educational support, and transitional services for youth exiting child protection services. Recently, they have also opened a youth emergency shelter. At-risk youth who have used YOU have found them useful, based on internal audit data. However, the rates of drug use, depression, and anxiety symptoms remain high compared to youth not at risk of homelessness, even after the provision of these services. This could be most likely due to the nature of the multiple challenges faced by this population.

The authors hypothesized that a further augmentation of current services offered by YOU would lead to an improvement in service user experiences and subsequent improvement in their mental health. The authors were keen to explore the feasibility of applying a standard breath-based yoga intervention coupled with social-emotional learning development based on positive findings in other study populations.

The assessed intervention was SKY Schools, formerly the Youth Empowerment Seminar (YES). SKY Schools is an evidence-based biopsychosocial program recognized by the Collaborative for Academic Social and Emotional Learning (CASEL), a program of the United Nations NGO the International Association for Human Values. SKY Schools has been offered in nonclinical settings to over 150,000 youth in North America and is registered in the USA and Canada as SKYSchools.org.

The SKY Schools intervention consists of a cognitive social-emotional learning (SEL) curriculum complemented by a series of yoga-based breathing techniques known as Sudarshan Kriya Yoga (SKY). By inducing physiological calm, SKY breathing techniques might enhance a youth’s ability to learn and execute SEL strategies taught, especially during periods of high stress when they are most needed.

SKY School’s SEL curriculum is delivered by certified instructors via an interactive, in-person, peer support, multimodal workshop. This workshop targets five core SEL areas: self-management, self-awareness, social awareness, relationship skills, and responsible decision-making [9]. Previous research demonstrates that such skill development enhances and promotes positive well-being and prosocial behaviors and reduces the onset of mental health difficulties [10].

Sudarshan Kriya Yoga (SKY) is a standardized series of 3 controlled yoga breath-based techniques including Sudarshan Kriya (described later). There is extensive research to suggest that SKY can offer a useful augmentation strategy to promote well-being and to treat various mental health conditions in the adult population including substance use, depression, anxiety, and PTSD [11]. Particularly, in two studies conducted on participants with substance use disorder, SKY was found to significantly improve quality of life and depression symptoms [12], mean age of control and SKY group was 37.0 ± 9.6 years and 39.2 ± 10.4 years, respectively; (*n* = 55) and control group (*n* = 29)). In another similar study in this population (*n* = 60), randomized equally to SKY or control with age ranges of 35.6 ± 8.1 and 37.8 ± 7.3 years, respectively [13], comparable results were found.

Data on adolescents and young adults, while limited, also shows promise. A randomized controlled trial in a multiethnic, nonclinical cohort of older adolescents (mean age 19.7) in the USA found that compared to a nonintervention control group (*n* = 47), SKY (*n* = 29) caused significant reductions in depression and stress; and significant improvements in mental health, positive affect, mindfulness, and social connectedness; as compared to two other evidence-based wellness programs, Foundations in Emotional Intelligence (*n* = 21) and Mindfulness-Based Stress Reduction (*n* = 34); which produced significant improvements in mindfulness only or no outcomes, respectively [14]. An open-label study (*n* = 59) of high school students (mean age 15.6 years; range 14–16 years) found significant improvements in the SEL constructs of self-awareness, self-management, relationship skills, and responsible decision-making [15]. Another publication reported on the combined findings of two non-randomized open-trial pilot studies on SKY conducted on a total of 74 young adults (age 25.4 ± 6.6 years; 55% female). Those who practiced SKY had a significant reduction in depression and stress and significant improvements in positive affect, emotion regulation, life satisfaction, and social connectedness [16]. Recent data from India from a reasonably large sample size of an experimental group (*n* = 237) of teenage students who had been practicing SKY for the past 1 year were compared with a control group (*n* = 218), who had not practiced any form of yoga or meditation. These cross-sectional survey data showed that the regular practice of SKY led to significant reductions in emotional, conduct, and peer problems as well as promoted prosocial behavior [17].

We examined the feasibility of providing SKY Schools intervention programs to at-risk youth samples in London, Ontario, Canada. We used a mixed methods approach to determine if a service user’s experiences can be used to further enhance the delivery of SKY Schools for this vulnerable population.

## Methods

### Study design

We conducted a mixed methods feasibility open-trial study, with a pre-post design, that explored delivering an 8-week rolling program of SKY Schools in at-risk youth. The study was approved by the Western University Health Sciences Research Ethics Board (approval # 107708) and registered at clinicaltrials.gov (NCT0274924).

### Study participants

**Fig. 1:**
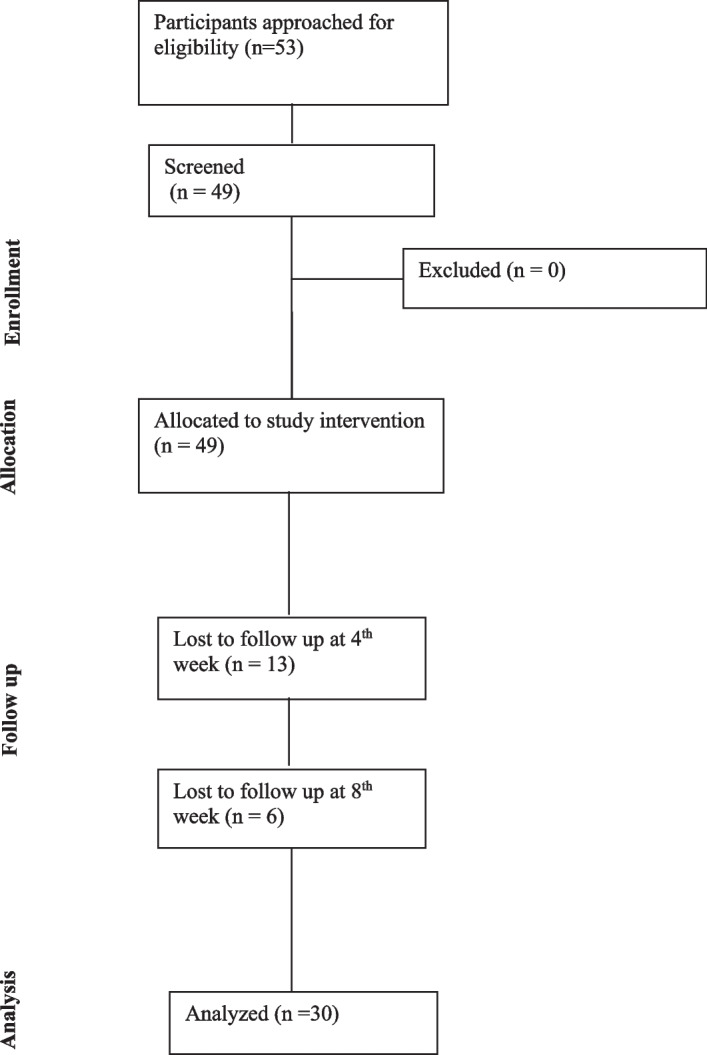
CONSORT diagram showing the flow of participants through screening to sample available for analysis

Potential participants were screened by research staff trained on Research, Ethics Compliance, and Safety offered by the Collaborative Institutional Training Initiative (CITI) Canada and the Lawson Health Research Institute. Informed consent was obtained from all the participants before beginning any study procedures. Inclusion criteria included youth aged 16 to 25 years who were either at risk of homelessness and/or in transition housing, had sufficient hearing to be able to follow verbal instructions, were able to sit without physical discomfort for 30 min, and were willing and able to attend all 4 daily initial SKY Schools training sessions and at least 5 of the 7 weekly follow-up sessions. Participants also needed to be willing to dedicate 20 min per day to Sudarshan Kriya Yoga (SKY) practice. Individuals were ineligible if they were currently participating in other similar studies or currently practicing any type of formal meditation, mindfulness, or breathing techniques regularly. There were no medication restrictions for the study. Recruitment was completed over 42 months between April 2016 and October 2019. A total of 53 participants were approached and 49 (92%) consented to participate (Table 1 and Fig. 1).

**Table 1:** Demographics of study participants at baseline (*n* = 49)

	*n*	%
Gender		
Female	26	53.1
Male	21	42.9
Other	2	4.1
Birth country		
Canada	22	44.9
Syria	15	30.6
Iraq	3	6.1
Libya	3	6.1
Other	5	10.0
First language		
English	26	53.1
Arabic	19	38.8
Kurdish	2	4.1
Napalese	1	2.0
Swahili	1	2.0
Employment status		
Unemployed	25	51.0
Student	20	40.8
Employed	3	6.1
Volunteer	1	2.0
Received treatment for substance use		
No	44	89.8
Yes	5	10.2
Ever been homeless		
No	23	44.9
Yes	22	46.9
Declined to answer	4	8.2

*n* = 49

### Sample size

No formal sample size power calculations were necessary for this feasibility study as the outcomes were the feasibility and acceptability of the intervention [18]. However, as this was also a pilot study, we a priori set the sample size to *n* = 30 as the final included sample, a commonly used sample size as suggested for pilot studies [19]. Based on our past clinical trial experience with this population and published literature, we a priori estimated a 50% attrition rate. Hence, we planned to recruit up to 60 participants to arrive at a final sample size of 30 participants.

### Procedures

Participants were recruited via study posters and information sessions offered at YOU, as well as key community locations such as libraries and community centers. Posters contained a brief description of the study and contact information for the research team. The information sessions provided individuals an opportunity to meet with members of the research team, learn more about the study, and ask any questions. Interested youth who contacted the research team or attended the information sessions were invited to attend an upcoming SKY Schools program. SKY Schools programs were held initially at YOU in 2016–2017, but due to inadequate recruitment (discussed later), we expanded to additional youth resource centers for refugee and migrant populations in 2018–2019. Trained raters met with participants at the community location where the SKY Schools intervention was held to complete an informed consent as well as collect baseline (week 0) information. Additionally, quantitative and qualitative data collection, as described below under Measures, occurred at weeks 4 and 8.

### Study intervention

The SKY intervention was offered over the duration of the study in 6 cohorts, the first 5 of which ranged in group size from *n* = 2–9 with a mean group size of 5.6. However, the final cohort enrolled 21 participants. The SKY Schools program was taught in two phases: (1) an active learning phase, which consisted of four consecutive days (3 h/day) of social-emotional learning (SEL) skills taught in a multimodal, interactive format interwoven with SKY training, and (2) a reinforcement phase. The SKY Schools learning phase is constructed around three major modules: Healthy Mind, Healthy Body, and Healthy Lifestyle.

The Healthy Mind Module includes stress management and relaxation techniques and is designed to foster a positive mental attitude as well as a calm state of mind. It utilizes a series of breath-based yoga breathing techniques, known as SKY. These breathing techniques include (a) Victory Breath (an advanced form of the yogic *Ujjayi* breath), (b) Bellow’s Breath (termed *Bhastrika* in yoga literature), and (c) *Sudarshan Kriya* (SK) or the Rhythmic Breath Technique (see Supplementary file for further details).

The Healthy Body Module includes a program of physical activities to increase flexibility and strengthen the body, along with interactive discussions and experiential processes to encourage mindful eating, and healthy nutrition choices, and recognize the impact of ingested substances on the body. The Healthy Lifestyle Module includes further interactive processes and dynamic discussions intended to foster the development of various skill sets such as emotion regulation and conflict negotiation, resistance skills, problem-solving, team building, relationship skills, and goal setting. The active learning phase was followed by a reinforcement phase that involved follow-up sessions either weekly (90 min each) or twice a week (45 min to 60 min each) for the subsequent 7 weeks.

Two certified instructors from SKY Schools delivered the program, under the supervision of their research director. After the initial 4-day training, participants were asked to practice SKY daily for 20–25 min in addition to attending the weekly follow-up sessions. Full meals were provided at the beginning or end of each training session of the active learning phase.

### Focus groups

Upon completion of the rolling SKY Schools intervention groups, all participants were invited to attend a 1-h focused group discussion (FGD) directly following the last follow-up session. FGDs are an effective tool in qualitative research allowing for informal interactions between participants and the facilitator that permit a better understanding of the participants’ viewpoints on SKY Schools [20]. During each FGD, a member of the research team followed an interview guide to facilitate discussions regarding the usefulness of the SKY Schools program and how it could be improved upon. The qualitative results of each FGD were used to provide insight into the feasibility of the program from the point-of-view of the participant, while also allowing the research team to evaluate the program and make changes, if necessary (e.g., changes in the duration of the course, topics covered), as well as the usefulness of the program in promoting social inclusion, improving mental health, and reducing substance use. All FGDs were audio recorded, field notes were collected, and recordings were later transcribed by a member of the research team. FGDs were carried out in a conversational style, starting with open-ended questions. Participants were later asked to identify what they liked and did not like about the program, what was helpful or harmful, and their challenges/barriers along with seeking their suggestions as to how the SKY Schools program could be improved upon. When feasible, the study staff implemented these suggestions in the next group itself. We planned to use this information to improve future SKY Schools interventions.

CAD $20 was offered to each study participant after each interview including the focus group. Hence, if a participant completed all parts of the study (week 0, week 4, week 8, and focus group), they received $80 in total. Additionally, the cost of public transportation to and from the intervention location was reimbursed upon submission of original bus stubs. Last, a nutritious snack or food voucher worth $10 was provided after each weekly reinforcement session.

### Measures

The following feasibility outcome measures were collected: (1) number of potential participants approached per month, (2) numbers (proportion) who were successfully screened, (3) proportion of screened participants who enrolled, (4) rate of retention in the study, (5) rate of adherence to study protocol, (6) proportion of planned ratings that were completed, (7) intervention cost per case, (8) completeness of final data for analysis, (9) length of time to collect all data, (10) quality of all collected data, (11) determining if YOU, Canadian Mental Health Association, and other similar community organizations were willing to conduct the study as per study protocol, (12) determining if there were any capacity issues with partners providing intervention and investigators being able to perform the tasks that they were committed to doing, (13) determining if there were any problems of entering the data into a computer, (14) preliminary data about the safety of the intervention, and (15) preliminary estimate of treatment effects using standard descriptive statistics such as means, proportions, confidence intervals and standard deviations on clinical rating scales to assess community integration (social inclusion), substance use, and mental health applied at weeks 0, 4, and 8.

The Community Integration Questionnaire (CIQ) consists of two subscales. The physical integration subscale assesses community involvement by asking participants to indicate “yes”, “no”, “do not know”, or “declined to participate” in any of the 7 community activities in the past month. The psychological integration subscale assesses the sense of belonging to where one lives and is assessed using a 5-point Likert scale ranging from strongly disagree (1 point) to strongly agree (5 points) for 4 questions on belongingness. The total score for the psychological integration subscale ranges from 4 to 20, with higher scores indicating a greater sense of belonging or psychological integration. Research has demonstrated adequate test–retest reliability and internal consistency [21] of the CIQ.

The Global Appraisal of Individual Needs–Substance Problem Scale (GAIN-SPS) measures the recency, breadth, and frequency of any substance use and associated problems. It consists of 13 questions, the first 7 of which request participants to indicate the last time substance use problems occurred with options including “past month”, “2–12 months ago”, “1 or more years ago”, “never”, “don’t know”, or “declined”. Scores are calculated by counting the number of problems that the participant experienced during the last year. Each symptom can be rated as either a 4, if they had a problem in the past month; a 3, if in the last 2 to 3 months; or a 2, if in the last 4 to 12 months. Hence, a higher score suggests a higher risk of substance use disorder. Questions 8 through 13 ask participants to provide specific ages for the onset of substance use, funds spent in the last month, and the number of days in the last month experiencing substance use problems. For the purposes of this study, researchers utilized a total past month score for questions 1–7 to assess changes in substance use from month to month. The GAIN-SPS has demonstrated good internal consistency and test–retest reliability [22].

The Colorado Symptom Index (CSI) assesses overall mental health and consists of 14 Likert scale questions with available options of “not at all” (score of 0), “once during the month”, “several times during the month”, “several times a week”, “at least every day” (score of 4), “do not know”, and “declined”. Total scores range from 0 to 56, with higher scores indicating more severe mental health symptoms including anxiety, depression, paranoia, suicidality, and thoughts of violence. The CSI has demonstrated excellent internal consistency, test–retest reliability, and validity across a range of scenarios [23].

At week 0, we asked participants to also complete an in-house created demographic, service, and housing history (DSSH) questionnaire [24]. Recreational drug use was not actively monitored, instead, the self-reported frequency of drug use was documented at each assessment using the GAIN-SPS.

### Data analysis (mixed-methods design)

The focus of this mixed-methods study was to (1) determine the feasibility of the protocol, and (2) collect qualitative information regarding the usefulness of the program from the youth’s point of view. The methods of analysis for each of the points mentioned above are described below. (1) Study success criteria: If we were able to (a) recruit one participant per week, (b) at least 60% of eligible participants were recruited, (c) retain at least 60% of those enrolled, and additionally if (d) 95% of the retained participants completed study questionnaires and attended focus groups/exit interviews, then we would have been successful. (2) The qualitative component of the study used a descriptive-exploratory method to explore the perspectives of the participants to better understand their experience of SKY Schools. This helped to identify what participants had liked and disliked about the program, and what might be done to improve future implementation. Data collection took the form of FGDs, in which open-ended questions were asked.

To understand the perspectives of participants, an inductive thematic analysis rooted in Braun and Clark’s six-phase framework for thematic analysis (i.e., becoming familiar with the data, generating initial codes, searching for themes, reviewing themes, defining the themes, and writing up) was completed on the transcribed data. Thematic analysis is a standard flexible method to support descriptive-explorative studies, by identifying underlying themes and patterns in the data [25].

Before commencing the analysis, to immerse themselves in the data, two team members independently read through the transcripts while listening to the audio recording to help find meaning and patterns within the data. These two members of the research team collaboratively performed open coding (i.e., developing and modifying codes as one worked through the data). Once this open coding was completed, the two researchers aggregated the codes and examined them for patterns to create groups and subthemes based on similarity of meaning. After assessing the identified themes for data coherence (within themes) and distinctions (between themes), the subthemes were grouped into major themes. After identifying the major themes, the research team met to discuss and draw conclusions.

## Summary of quantitative and qualitative findings

The statistical analysis plan allowed data collection from multiple sources including changes in quantitative data from participants over the 8-week period and comparisons with participant-level qualitative data from open focus group discussions. We believe that this method led to adequate triangulation, an important technique of amalgamating quantitative and qualitative findings, as suggested by Patton [26].

## Results

### Primary outcomes (feasibility)

We planned to consent up to 60 participants with the aim of retaining at least 30 until study completion. This outcome was achieved. However, one of our study success criteria was for the study team to recruit one study participant per week. This criterion was not met as 49 participants were recruited over a duration of 182 weeks (April 2016 to October 2019, recruitment rate of 0.27 participants per week).

A total of 53 potential participants were approached over a period of 42 months, which gives an average of 1.26 potential participants approached per month. Of those approached, *n* = 49 (92.5%) were screened, 100% of which met the study inclusion criteria and were hence successfully enrolled (*n* = 49). Of those enrolled, 61.2% (*n* = 30) were retained until study completion. Thirteen (26.5%) withdrew prior to the fourth week of the study intervention and 6 withdrew (12.2%) prior to the eighth week of the study intervention (see Fig. 1: CONSORT diagram and Table 3). Of the 49 enrolled participants, questionnaires were completed by 48 (98.0%) participants at week 0, 34 (69.4%) at week 4, and 23 (47.0%) at week 8 (see further details in Table 3).

**Fig. 2:**
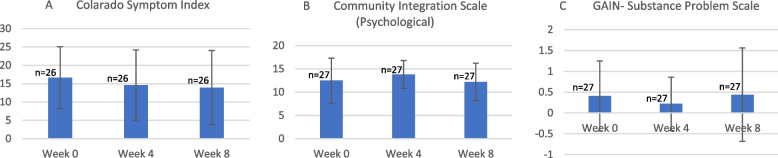
Change in Mean Scores over eight weeks from all completer’s participant data. Bar charts represent mean ± Standard Deviation. Data was collected on three self rated scales for A: mental health using the Colorado Symptom Index (CSI), B: sense of belongingness to the community using the Community Integration Scale (CIS)-Psychological Subscale and C: recency, frequency and breadth of any substance use, the Global Appraisal of Individual Needs-Substance Problem Scale (GAIN-SPS). Higher scores on the A) CSI represents higher degree of mental health difficulties, B) CIS represents higher degree of belongingness, and C) GAIN-SPS represents higher degree of substance use problems

Of the participants enrolled, 20 were immigrants and were identified as being at risk of homelessness due to the vulnerability of this population [27]. Computer-based data entry was successfully completed with 100% of collected data being entered into the study database. Quantitative data of participants who completed the duration of the study are represented graphically in Fig. 2. The data trend suggests that over a period of 8 weeks, the SKY Schools intervention led to an improvement in mental health as per the Colorado Symptom Index, a nonsustained improvement in community integration on the psychological subscale, and an improvement in substance use at week 4 which was also not sustained at the 8-week data collection point.

Two minor protocol deviations were noted during the conduct of the study. Weekly follow-ups were implemented rather than the planned semi-weekly follow-ups. We also offered a snack gift card to the participants in the later part of the study, rather than an actual snack. These changes were made based on suggestions gathered through participant feedback during the initial focus groups. The calculated cost of the intervention per participant was CAD $176.20.

The Youth Opportunities Unlimited (YOU) community organization, our initial recruiting partner, was willing to conduct the study; however, it was felt that they were unable to provide consistent support to participants and the team. Hence, we determined that it would not be possible to meet recruitment goals from this organization alone. To this end, we approached two other community organizations including the London Intercommunity Health Centre (LIHC) and the Canadian Mental Health Association (CMHA). The LIHC and CMHA turned out to be enthusiastic partners, working with the study team to provide available appropriate space during a time/date that was felt to be more convenient for participants. The facilitators of the SKY Schools program were able to successfully deliver the intervention; however, during the focus groups, participants informed study staff that on a few occasions, the intervention facilitators arrived late and in one instance failed to adequately communicate to the study staff and participants that a follow-up SKY Schools session had been rescheduled. Thirty participants attended focus groups, with group sizes ranging from *n* = 2–9 and a mean group size of *n* = 6.

No serious adverse events (SAEs) were reported during the study. Reported adverse effects, by a single participant, included the following (a) feeling as if one could not move their hands, (b) a sensation of ants walking on the skin, (c) being uncomfortable while laying on a yoga mat, (d) a headache, and (f) muscle soreness. No other adverse events were reported. This study did not utilize a formal method of collecting adverse events, rather questions were posed by the study staff during the focus groups asking each participant to report any perceived harms associated with participating in SKY Schools.

### Qualitative outcomes (focus group)

Nine major themes and 42 corresponding subthemes were identified from the analysis of the focus group transcripts (Table 2). Qualitative results were found to be overall cohesive with the quantitative results. During focus groups, participants shared that they were aware that some participants had dropped out of the intervention, and they identified what they believed were the reasons for withdrawals. In terms of their own perceived effects of the intervention, some participants expressed an improvement in emotional regulation while others did not, and some enjoyed the social aspect of the program while others felt this needed to be improved. This is consistent with the quantitative findings of a 61% retention rate, some improvement in mental health symptoms, a nonsustained improvement in substance use, and an unchanged community integration.

**Table 2:** Focus group themes and sub-themes

Major themes	Sub-themes
Personal investment	Varying participation
	Dwindling participation
	Progressive commitment
Inadequate coordination	Improved consistency needed
	Need to start on time
	Timing is critical
	Misinformed on support
	Proactive resolutions needed
Underwhelming experience	No improvement
	Tepid attitude toward SKY Schools
	Stagnant growth
Rewarding experience	Positive response to SKY Schools
	Better than anticipated
	Practical
	Improved lifestyle
Improved emotional adaptability	Relaxing technique
	Restorative
	Enhanced emotional regulation
	Improved focus
	Increased resiliency
	SKY Schools as a coping mechanism
Interpersonal dynamics	Supportive community (change to Social facilitation)
	Meet and greet needed
	Social dining suggested
	Lack of trust
	Existing peer relationships
	Influence of gender
Facilitator influence	Accessible facilitators
	Supportive facilitators
	Insufficient support
	Overenthusiastic instructors (change to facilitators)
	Participant accountability
	Program discipline required
	Lax instructors (change to lax facilitators)
	Rigid dietary options
Structural suggestions	Improvements to structure
	Appropriate session duration
	Disruptive transition
Suggested enhancements	Environmental improvements
	Valuing extrinsic benefits
	Good location
	Adverse effects

The nine major themes and subthemes are detailed below.

### Rewarding experience

Many participants found SKY Schools to be a helpful technique that led to an improved lifestyle and sense of well-being. They found the techniques easy to learn and felt they were a practical tool to establish in their lives. Participants provided a positive response to SKY Schools, indicated that it was better than anticipated, found it to be practical, and reported an improved lifestyle:

“For me personally I think it was better than I thought it was going to be just because there was such a wide variety of techniques; it wasn’t just focusing on yoga or the mindfulness or the breathing and it kind of encompassed everything, which was nice. So each strategy so to speak, I feel you could either you could do any of those individually and they would all be effective but because we were doing all of them, I felt it bettered the results, and I also felt that the home practice was something easy enough to remember, the order of the movements and the breathing techniques and it wasn’t like it took two hours to do or something. It’s very functional in day-to-day life”.

### Improved emotional adaptability

Participants reported multiple emotional benefits of SKY Schools including a sense of relaxation and restoration, improved emotional regulation, and improved focus. Participants had a sense of vitality after attending SKY Schools and felt it could be used to cope with daily stressors. Participants indicated that SKY Schools would help them to focus and stay in the present moment:

“It was just relaxing, soothing. I've never done meditation or yoga before. So I was very optimistic, not optimistic, whatever the opposite of optimistic is, I was that I'm just like oh this is going to be ridiculous... yeah skeptical... And I didn't think I was even going to be interested, but after a couple of days...I felt good, felt energized, I felt relaxed...This is a weird hobby of mine, but I go to a graveyard that's already negative. I go to a graveyard and I don't really go to the graveyard to visit anybody. I go there cause there's deer. And then if I can get close to a deer, it feels really peaceful. And you feel like closer to nature, but then as soon as you step outside of that graveyard, you start seeing traffic. You get that feeling like, Oh man, it's time to go back to life. You know? But with the meditation it felt like I was in a forest with deer relaxed, but then when I left, I was like, okay, now it's time to go back to life, but I probably don't want to do that...”

### Interpersonal dynamics

Participants appreciated the atmosphere of the group and the social facilitation it provided, reporting that it made them feel more comfortable trying something new, among their supportive peers. They expressed that they would have preferred to have a more structured interaction such as a “meet and greet” and social dining. Participants felt that there was an optimal level of familiarity with their group that would increase comfort without overshadowing the purpose of the program. They identified preexisting peer relationships as being both helpful and distracting. Participants indicated that gender played a role in their experience in the SKY Schools program and their comfort was increased by the presence of both a male and female facilitator. Study participants were diverse and joined the study with a large spectrum of experiences resulting in different preexisting levels of trust, including some participants who indicated they lacked trust in others. This preexisting ability to share and be open, or not, led participants to have different experiences:

“Personally, it takes me a long time to be open and trust people so like actually being able to go and say, ‘well I’m having these problems’ was an issue for me because it’s like talk talking about things that you would talk to a counselor about and then these people you don’t really know anything about. So, it was kind of challenging regardless of if it was one day, two days, it wouldn’t really have made a difference.”

### Personal investment

Participants expressed that the perceived outcome of participating in SKY Schools was proportional to their own level of involvement. They identified that there was a varying level of participation between participants, dwindling participation, and a progressive commitment from participants who were retained in the study:

“I think it [number of withdrawn participants] has less to do with the program... I think it has very little to do with how this program is structured. I think it has everything to do with...the types of kids that are involved and then it doesn't matter what it is at that point...I don't think that focusing on how to change it is going to benefit. I think, no matter how it's structured, you're still gonna have people drop off over time... I think that's why a lot of people don't show up because... it [SKY Schools Program] doesn't have any place in the minds, in the greater context of what you're doing”.

### Underwhelming or negative

Participants provided a varied view of their experience in the SKY Schools program. Some participants indicated that no improvement was seen, while others had a tepid attitude toward SKY Schools and indicated that their personal growth was stagnant. A few identified facilitators were too overenthusiastic which was off-putting:

“I personally didn’t really get a lot of benefits from it [SKY Schools]. I did it for about two months and then stopped using it because I wasn’t noticing any differences. I do a general meditation practice on my own that I’ve been doing for like 10 years so I find that more helpful for me.”

### Inadequate coordination

Participants indicated that there were logistical issues that caused significant barriers to attending SKY Schools regularly. These included things such as transportation, time of day for the program, childcare, and communication about the program itself. Participants felt they were not provided with an adequate process for reporting and escalating logistical program issues. Participants suggested that improved consistency was needed, the program should start on time, the scheduled time of day for SKY Schools was critical, they were misinformed on the available support, and proactive resolutions were needed:

“[Having kids around] was distracting to me too. I felt uncomfortable telling them 'you guys told me I could bring my children and then you would watch them.' And that’s why I didn’t want to bring my son because I feel it would be more of a distraction. I understand that the childcare is needed and makes sense but maybe if they had a way to find a better block out the window or try and just soundproof the situation.”

### Facilitator influence

Participants believed they would have further benefited from a stricter discipline-based approach. While they identified that participants should be accountable for their own behavior, they also perceived that facilitators were lax in applying discipline. However, they also expressed that the facilitators were largely accessible and supportive:

“It's [SKY Schools] something different as I was already trying to learn meditation with many different points of view. Whereas I was struggling a lot before or trying to learn stuff from the video and trying to learn stuff from just books and videos, this one separate thing. Then being able to just learn personally from somebody... an expert and that I think is the biggest difference for me...That's really what kept me coming back. Cause I mean, I can sit at home and wonder the entire time during this right, I can’t ask questions or anything like that, just being able to be right there. I think that I was already looking for meditation stuff before the course was offered. So it's almost [auspicious] at the same time. And just to be able to have the opportunity to do a free, you know, to some even certain extent more structured, you know. Your classmates, not just the yoga, it's not just the meditation. It was kind of like a little bit more of a different take on that. So I did enjoy that aspect a lot.”

### Structural suggestions

Participants provided feedback on further enhancements for the program including suggestions for improvements to the structure and session duration and reducing disruptive transitions between portions of the SKY Schools program:

“If you had more of not such a big span between [SKY Schools sessions]. If you do like twice a week, because when you do it once a week, there's just that huge time [between sessions]. … But the one thing I found was that with that huge gap in between each session [before the next] follow-up, it was almost too big of one because people would forget. And especially that it was on Monday... Just coming out of the weekend...That's the thing that would really throw people off... Tuesday, Thursday, something during the week, like Monday, you're recovering...But if you did it twice a week, rather than only once a week, I think it would be good. Cause at the beginning, it was consistent. Everybody showed up and I was just like, Oh man, we got quite a group here. It felt more comfortable. I would say either twice a week or try and not do it on the Monday.”

### Suggested enhancements

Participants indicated that they would have enjoyed the SKY Schools program more with some environmental improvements, such as a warmer room. Participants suggested that the location of the program was very important and provided feedback on adverse events including discomfort related to the environment. They identified the value of extrinsic benefits to study participation including study compensation:

“Eventually we started doing the Tims (reference to Tim Hortons, a large chain of Canadian café) cards. I found that to be better. For transportation, I just walk so... going [with] the gift cards to the restaurant there wasn’t a lot of things you can get for that...And the Tims card, like you had the option of getting what you wanted umm as well so if somebody didn’t like a particular food item or couldn’t eat a particular food item, you wouldn’t have to worry about that.”

## Discussion

The study success criteria, as established a priori, were met for participant retention and questionnaire completion. However, this study failed to recruit participants at the planned recruitment rate of 1 participant per week, rather we recruited 1.26 participants per month. Our poor recruitment rate likely reflects the nature of the underlying study population. It has been previously demonstrated that it is hard to recruit participants from this population as they are generally found to be distrustful of research studies, feel withdrawn, or are concerned with their own difficulties associated with being homeless or at risk of homelessness [28]. This study recruited participants from London, Ontario only, which as of July 14, 2021, was reported to have 1278 people who identified themselves as homeless [29], 20% of whom are expected to be youth [30]. It may be that to achieve a recruitment rate of 1 participant per week from this population, a future study will need to expand recruitment outside of London. Additionally, it will be important to focus recruitment on potential participants who are at risk of homelessness rather than those who are in transitional housing such as those housed at YOU.

A total of 6 cohorts were used in the study. The first 5 cohorts had a group size between 2 and 9, with cohort 6 having a group size of *n* = 21. Twenty participants were immigrants and were identified as being at risk due to the vulnerable nature of this population [27]. Recruitment and retention rates from the final cohort may have been different than the initial cohorts due to the difference in recruitment source (initial cohorts were recruited from YOU). It is likely that the results from our study are heterogeneous, as they include two separate populations: youth at risk of homelessness who are local to the city and a newer immigrant population who have different needs and aspirations.

This mixed methods study utilized focus groups to identify what participants liked or did not like about the SKY Schools program and to elucidate how the program might be further improved in a future study. Of those who characterized their overall response to YES as positive, there was an appreciation of the various components, both the mind–body and cognitive aspects of SKY Schools. Some participants suggested that their experience of SKY Schools was “better than expected” and felt that the fact that it drew from multiple approaches enhanced their experience. Other positive characteristics that participants mentioned were that SKY Schools are practical, and easily integrated into their lives, due to their lack of equipment and ability to be practiced anywhere at any time. It may be possible that participants view the techniques learned through SKY Schools as having reasonable demands given the associated benefits. Another benefit that satisfied participants reported was improved lifestyle habits. These participants thought that the structure of SKY Schools enabled them to create structure in other domains of their lives.

Participants reported experiencing relaxation during SKY Schools, and increasingly in their lives as a result of SKY Schools. They reported that the experience of relaxation was most comparable to a sleep state and that this relaxation was advantageous for their emotional well-being. In terms of enhanced emotional well-being, participants found it easier to navigate their emotions after attending the SKY Schools intervention.

In terms of the negative perception of the intervention, under the theme of inadequate coordination, participants highlighted the importance of their expectations of consistency and punctuality, which may be essential to maintaining and building trust in a provider [31]. After the completion of the study, we elicited retrospective feedback from the SKY Schools facilitators regarding this reported concern of lack of punctuality. The facilitators reported extreme weather impacting their travel during the winter months and scheduling difficulties as the main reasons for some instances of lack of punctuality. Future studies should consider addressing and managing these difficulties.

Another contributor to the perception of inadequate coordination was perceived misinformation about available support such as childcare. Despite the lack of any information regarding the provision of childcare during the informed consent process, it appears that this support was indeed a perception among the participants. It could be that over the duration of the study, research staff, partnering agencies, and/or other co-participants could have offered this wrong information. This misinformation was unfortunate. Nevertheless, future studies should consider the provision of childcare as an additional support system to retain participants.

Enhancements suggested by the participants indicated that they were attuned to the sensory environment of the intervention. Participants suggested adding pleasing sounds, and ways to avoid aversive sensory stimulation, such as smell. Temperature was also identified as an important factor. Future research is needed to elucidate the exact role of sensory environments in youth development, but in other populations, such as young children with autism, the quality of the sensory environment is an important determinant of engagement with activities [32].

Another suggested enhancement included using a central location for the delivery of the intervention. This may have been more convenient for a larger number of participants. The participants also reported valuing the extrinsic benefits of the program, such as food or money. This is echoed in other behavior change research that extrinsic benefits are valuable insofar as they do not overwhelm intrinsic motivation [33]. This may also have the effect of attenuating stress associated with food or resource instability.

The participants also described how the response to the program may be moderated by personal characteristics. They thought this led to differences in personal commitment that changed over time. Some participants also thought that this may be moderated by placing the intervention in the wider social context of participants’ lives. In terms of identity, participants described that participation in the intervention sometimes interfered with other roles or occupational activities. Prompting youth to think about their existing identities or commitments and thinking of ways to integrate the intervention with the other domains of their life may be helpful.

The way that participants characterized their overall responses varied, from extremely positive to indifferent. Participants indicated that participation in SKY Schools led to an improved lifestyle, helping them to feel relaxed and restored after practicing the SKY techniques. However, other participants revealed a tepid attitude, as well as the explanation that they had participated in the program because of its novelty. A third participant’s perspective was that the intervention offered “no change”. Future research on the psychological perspectives or expectancies of participants at the beginning of the program may be helpful in investigating what kind of changes youth may have expected from the program. Participants with a tepid response to SKY Schools also mentioned that they thought that the response to SKY Schools would be moderated mostly by individual factors and that they would recommend SKY Schools only with knowledge of personal characteristics, as opposed to a global endorsement of the program. One participant felt that the intervention was overly monotonous and lacked the differentiation necessary to advance their skills.

It may be that the SKY Schools program is not appropriate for youth who are currently homeless. These youth may be too worried about finding their next meal and a place to sleep to focus on the program. It may be that youth who are currently using illicit drugs may not benefit from the SKY Schools intervention, which requires one to be not only physically present but mentally present.

Participants described three major themes associated with the social environment. They described the integral role of the facilitators in SKY Schools and the need for inclusivity in terms of facilitators. Participants reported that they thought having facilitators who shared the same gender they identified with was beneficial to learning. Existing peer relationships were important and future studies should continue to manage this in ways that may be further beneficial for participants. Participants suggested improving social dynamics by facilitating familiarity. Facilitators were also important because they played a crucial role in helping youth learn the techniques associated with the program. Participants reported feeling as though the experience was enhanced through the increased availability and willingness of facilitators to answer questions. However, overcontrol or over-enthusiasm by instructors was received negatively by participants.

This study was not designed to definitively demonstrate the clinical efficacy of the SKY Schools program but rather to assess if it is feasible to conduct a definitive study. The high level of questionnaire completion by retained participants, the completion of computer-based data entry, and the completion of statistical analyses of quantitative data demonstrated that it is feasible to collect data in this study population for demographics, mental health symptoms, substance use, and community involvement. Trend analyses of quantitative data suggest beneficial effects of SKY School’s mental health symptoms and at least an initial decrease in substance use. These findings need to be replicated in a larger randomized controlled trial (Table 3).

**Table 3:** Results of primary (feasibility) outcome measures collected

Recruitment duration of study = 42 months (April 2016 to October 2019)	*n* or proportion	Comments, if any				
Number of potential participants approached (total *N* = 53)	1.26 participants per month	There was a reduced rate of recruitment from our main partner, YOU, after the first year of recruitment. We later expanded our recruitment to LIHC and CMHA to complete the study				
Number (proportion) of participants who were successfully screened (total *N* = 49)	49/53 (92%)	None				
Number (proportion) of successfully screened participants who enrolled	49/49 (100%)	None				
Rate of participant retention	30/49 (61.2%)	There was no study intervention related serious adverse effects. 13 participants dropped off at 4 weeks and an additional 6 dropped off at 8 weeks for unknown reasons (lost to follow-up)				
Rate of adherence to study protocol	2 minor deviations	We offered once a week SKY follow-up rather than the planned twice weekly We gave a snack gift card rather than an actual snack Both these deviations were implemented based on participant feedback after first FGD				
Proportion of planned ratings that were completed	Range 90% to 100%	Measure	Week 0		Week 4	Week 8
		CSI	47/49		34/36	29/30
		CIS	48/49		34/36	29/30
		GAIN-SPS	48/49		36/36	27/30
		Range of available data	96%-98%		94%-100%	90%-97%
Intervention cost per case	CAD $176.2	Cost for delivery of intervention only				
Completeness of final data for analysis	NA	One participant data sheet was deemed lost at week 0. On all other available data sheets, self-rated scores were legible and analyzable				
Length of time to collect all data	NA	Data collection took place over a period of 47 months from January 11, 2016, to December 11, 2019				
Quality of all collected data	NA	There were no shortcomings				
Determining if YOU, Canadian Mental Health Association and other similar community organizations were willing to conduct the study as per study protocol	NA	See further details in the results section				
Determining if there were any capacity issues with partners providing intervention and investigators being able to perform the tasks that they were committed to doing	NA	See further details in the results section				
Determining if there were any problems of entering the data into a computer	NA	None				
Preliminary data about the safety of the intervention	1	One participant expressed minor side effects, see further details in the results section				

*YOU* Youth Opportunities Unlimited, *LIHC* London Intercommunity Health Centre, *CMHA* Canadian Mental Health Association, *SKY* Sudarshan Kriya Yoga, *FGD* Focus Group Discussion, *CSI* Colorado Symptom Index, *CIS* Community Integration Scale, *GAIN-SPS* Global Appraisal of Individual Needs-Substance Problem Scale, *CAD* Canadian Dollars

## Conclusions

### Limitations

This study did not use a formal scale for the collection of adverse events but rather relied on participant descriptive reports. To our knowledge, no validated scale for the assessment of adverse events in yoga interventions has been published to date. Attrition in this study was high with 38.8% of participants withdrawing from the study intervention prior to the week 8 follow-up visit.

## Implications and future directions

Our findings confirm that the youth at risk of homelessness are a very difficult population to recruit and retain for the purpose of running a clinical trial. For a future study of this population, it would be imperative to have a multi-site study to recruit and retain at a more reliable rate. Second, while the participants offered a substantial number of suggestions to improve the delivery and content of the intervention, there were several critical observations. We believe that some of their negative biases toward the intervention could be based on their own experiences with the social aspect of the risk of homelessness. This might have led them to have poor self-esteem, distrust of the research staff and the SKY Schools facilitators, and concern with their own difficulties, as previously suggested by Hough et al. [28].

However, certain improvements may be feasible, such as providing a comfortable space (warm temperature and soft yoga mats) to practice SKY Schools, and at a time and location that is convenient for participants. Planning socialization time to accompany the SKY Schools program in the format of a “meet and greet” and social dining may help participants build trust with facilitators and other members of their SKY Schools group. Greater communication about what participants can expect in a future study as well as to whom they should bring their concerns or seek support from could further improve upon the experience of participants. Including facilitators of multiple genders for each program is also seen as important in providing a comfortable and safe environment for participants. Overall, participants indicated that they were satisfied with the content of the SKY Schools program, and most suggestions were related to administrative aspects of the study intervention. These administrative issues could readily be resolved with suggestions provided by participants and easily implemented in a future study. If, in the future, an appropriate validated scale is published for the assessment of adverse events in mind–body interventions, such a scale should be utilized.

## Summary

In summary, this feasibility study demonstrated that while it was difficult to recruit and retain youth who are homeless or at risk of homelessness for participation in SKY Schools, a future study might benefit from having a multi-site recruitment strategy. It was possible to collect all a priori-selected clinical outcome data from this population.

### Supplementary Information

**Additional file 1: Supplementary Table 1a.** Results of a repeated measures analysis descriptive statistics for available participant data. **Supplementary Table 1b.** Results of a repeated measures analysis for available participant data. **Supplementary Table 2.** Results of a Friedman Test for available participant data on the Community Integration Scale-Physical Integration. d.f. degrees of freedom. Md: 50th (Median).

## Data Availability

The datasets used and/or analyzed during the current study are available from the corresponding author upon reasonable request.
