# Supplementary material for: A feasibility (pilot) mixed methods study of an innovative non-pharmacological breath-based yoga and social-emotional intervention program in an at-risk youth sample in London, Canada
Source: Pilot Feasibility Stud. 2024 Feb 7;10:26. doi: 10.1186/s40814-024-01452-0 (PMC10848429; doi:10.1186/s40814-024-01452-0)
Supplement: Supplementary file 1 — Additional file 1: Supplementary Table 1a. Results of a repeated measures analysis descriptive statistics for available participant data. Supplementary Table 1b. Results of a repeated measures analysis for available participant data. Supplementary Table 2. Results of a Friedman Test for available participant data on the Community Integration Scale-Physical Integration. d.f. degrees of freedom. Md: 50th (Median). [file 40814_2024_1452_MOESM1_ESM.docx]

**A feasibility (pilot) mixed methods study of an innovative non-pharmacological breath -based yoga and social emotional learning intervention program in an at-risk youth sample in London, Canada.**

**Supplementary File**

**SKY Schools program**

The study intervention, developed by the charitable and non-profit International Association for Human Values and taught to over 150,000 youths in North America, is SKY Schools (previously named Youth Empowerment Seminar YES!). This age appropriate bio-psycho-social program consists of a cognitive SEL curriculum complemented by a series of yoga-based breathing techniques, known as Sudarshan Kriya Yoga (SKY). Social emotional learning (SEL) skills help people understand and manage their emotions, build successful relationships, and deal with life stressors/events in a positive and constructive way. There are five core SEL areas: self-management, self-awareness, social awareness, relationship skills and responsible decision-making. Through games, activities, and interactive discussions, the program is designed to strengthen youth’s skills in each of these areas. SKY is a standardized collection of yogic-breathing techniques which includes: (a) Victory Breath: an advanced form of Yogic Breathing practice called Ujjayi Breath, (b) Bellow's Breath (also referred to as Bhastrika) and (c) Sudarshan Kriya (SK) or the Rhythmic Breath Technique.

Victory Breath is created by a slight voluntary contraction of the laryngeal muscles with a partial closure of the glottis. This creates increased airway resistance and enables control of the rate of air flow. The advanced form of Victory Breath practiced in SKY utilizes three distinct arm postures, and specific ratios of inhalation, exhalation, and breath-holds. Victory Breath consists of 4 to 6 breaths per minute. Bellow’s Breath (Bhastrika) involves forceful rapid breathing through the nose at a rate of 20 to 30 breaths per minute. The use of the arm movements leads to an increase in the force as well as depth of each respiration. SK involves rhythmic, cyclical breathing in which there are no pauses between inhalation and exhalation. SK involves multiple rounds of slow (15-30 respiratory cycles per minute), medium (40-50 respiratory cycles per minute), and fast (60-100 cycles per minute) cycles with varying rhythms and durations that are standardised.

**Quantitative Results**

|  |  | n = | Mean | Standard Deviation |
| --- | --- | --- | --- | --- |
| Colorado Symptom Index | Week 0 | 26 | 16.62 | 8.45 |
|  | Week 4 | 26 | 14.62 | 9.57 |
|  | Week 8 | 26 | 13.92 | 10.10 |
| Global Appraisal of Individual Needs-Substance Problem Scale | Week 0 | 27 | 0.41 | 0.84 |
|  | Week 4 | 27 | 0.22 | 0.64 |
|  | Week 8 | 27 | 0.44 | 1.12 |
| Community Integration Scale  – Psychological Integration | Week 0 | 27 | 12.48 | 4.82 |
|  | Week 4 | 27 | 13.81 | 3.00 |
|  | Week 8 | 27 | 12.22 | 4.02 |

**Supplementary Table 1a.** Results of a repeated measures analysis descriptive statistics for available participant data.

|  | d.f. | F | p = | Multivariate partial eta squared | Effect size interpretation |
| --- | --- | --- | --- | --- | --- |
| Colorado Symptom Index | 2, 24 | 1.08 | 0.357 | 0.082 | moderate |
| Gain Substance Problem Scale  past month* | 1.33, 34.58 | 1.05 | 0.338 | 0.038 | small |
| Community Integration Scale – Psychological Integration | 2, 26 | 1.18 | 0.317 | 0.043 | small |

**Supplementary Table 1b.** Results of a repeated measures analysis for available participant data.

d.f.: degrees of freedom

*Mauchly’s Test of Sphericity violated, Greenhouse-Geisser correction used

| In the past month have you? | d.f. | n = | Chi-Square | P = | Week 0 Md | Week 4 Md | Week 8 Md | Kendall’s W | Effect size interpretation |
| --- | --- | --- | --- | --- | --- | --- | --- | --- | --- |
| Attended a movie or concert? | 2 | 33 | 2.21 | 0.331 | No | No | No | 0.033 | small |
| Participated in outside sports or recreation? | 2 | 33 | 1.29 | 0.526 | Yes | Yes | Yes | 0.019 | small |
| Gone to meet people at a restaurant or coffee shop? | 2 | 33 | 0.62 | 0.735 | Yes | Yes | Yes | 0.009 | small |
| Participated in a community event? | 2 | 33 | 2.00 | 0.368 | Yes | No | Yes | 0.030 | small |
| Gone to a place of worship or participated in a spiritual ceremony? | 2 | 33 | 2.65 | 0.266 | No | No | No | 0.040 | small |
| Participated in a volunteer activity? | 2 | 33 | 1.75 | 0.417 | No | Yes | No | 0.027 | small |
| Gone to a library? | 2 | 33 | 1.53 | 0.465 | Yes | Yes | Yes | 0.023 | small |

**Supplementary Table 2.** Results of a Friedman Test for available participant data on the Community Integration Scale-Physical Integration.

d.f. degrees of freedom

Md: 50^th^ (Median)
